# Identification of *Ulocladium chartarum* as an important indoor allergen source

**DOI:** 10.1111/all.14999

**Published:** 2021-07-28

**Authors:** Sandra Pfeiffer, Peter Sandler, Marianne Raith, Mariona Pascal, Rosa Maria Munoz‐Cano, Clara San Bartolome, Katharina Nöbauer, Santiago Quirce, Ebrahim Razzazi‐Fazeli, Margarete Focke‐Tejkl, Katja Sterflinger, Ines Swoboda

**Affiliations:** ^1^ The Molecular Biotechnology Section FH Campus Wien Campus Vienna Biocenter University of Applied Sciences Vienna Austria; ^2^ Immunology Department CDB Hospital Clinic de Barcelona; IDIBAPS University of Barcelona Barcelona Spain; ^3^ Spanish Network for Allergy – RETIC de Asma, Reacciones adversas y Alérgicas (ARADYAL Madrid Spain; ^4^ Hospital Clinic de Barcelona, Allergy Unit IDIBAPS University of Barcelona, ARADyAL Barcelona Spain; ^5^ VetCORE Facility for Research University of Veterinary Medicine Vienna Austria; ^6^ Department of Allergy La Paz University Hospital IdiPAZ Universidad Autonoma de Madrid Madrid Spain; ^7^ Division of Immunopathology Department of Pathophysiology and Allergy Research Center for Pathophysiology, Infectiology and Immunology Medical University of Vienna Vienna Austria; ^8^ Institute of Natural Sciences and Technology in the Arts Academy of Fine Arts Vienna Vienna Austria; ^9^ Present address: Business segment for Medicine and Medical Devices Agency for Health and Food Safety (AGES) Vienna Austria

**Keywords:** allergy diagnosis, indoor allergens, mold allergy, recombinant allergens, ulocladium chartarum

To the Editor,

The constant exposure to fungal spores, which constitute the largest proportion of aerobiological particles, can cause severe health problems, including allergic diseases.^1^, ^2^ Appropriate management of fungal allergies is hampered by the fact that our knowledge about fungal allergy is still limited to a small number of thoroughly investigated fungi, whereas for the majority of species, it is not yet known whether they play a role in allergic diseases.^2^ This lack of knowledge together with unreliable diagnostic results obtained with fungal allergen extracts of poor quality contributes to a general underdiagnosis of fungal allergy.^3^, ^4^ In the present study, we investigated the allergenic potential of *Ulocladium chartarum*, an opportunistic human pathogen^5^ that can grow on various substrates.^6^, ^7^ Exposure to *Ulocladium* species often occurs in the indoor environment as they are commonly detected inside damp buildings and are regarded as an indicator of water damages.^6^, ^7^, ^8^, ^9^ The fact that *U. chartarum* belongs to the same family as the outdoor aeroallergen source *Alternaria alternata* suggests that *U. chartarum* might also represent an allergen source.^6^, ^7^

IgE immunoblots, performed with sera from patients sensitized to different mold species (Table S1), demonstrated the high allergenic potential of *U. chartarum*, since patients' IgE antibodies recognized several *U. chartarum* proteins (Figure 1A), whereas exposure of the blotted proteins to sera from non‐allergic individuals or to immunodetection reagent did not lead to any unspecific binding (data not shown). Interestingly, despite their phylogenetic relationship, the IgE‐binding profile of *A. alternata* (Figure S1) differed significantly from the one of *U. chartarum*. Mold‐allergic patients not only recognized more proteins in *U. chartarum* than in *A. alternata* extract, but also showed an overall stronger reactivity to them.

**FIGURE 1:**
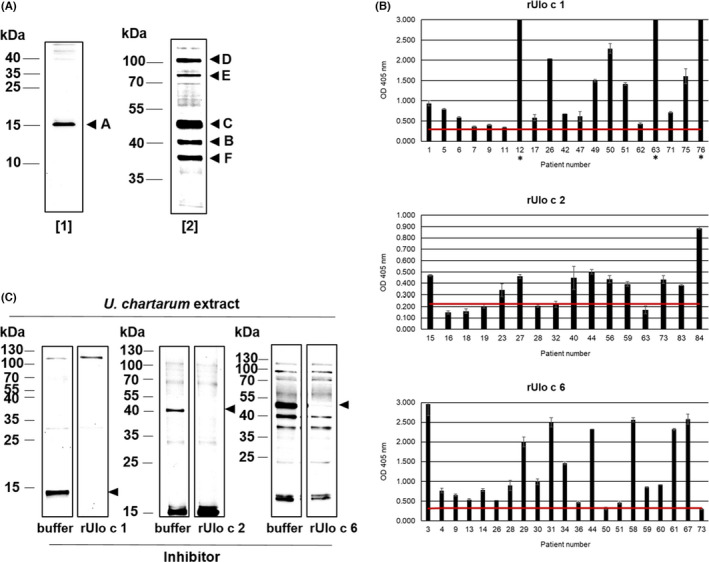
Identification and characterization of IgE‐reactive proteins from *Ulocladium chartarum*. (A) Nitrocellulose‐blotted proteins from *U. chartarum* were exposed to two pools of sera [pool 1: sera 1, 2, 45–47, and 74; pool 2: sera 3–4, 13–14, 28–31, 34–36, and 58–61] from mold‐allergic patients. IgE‐reactive proteins identified by mass spectrometry are marked with A, B, and C; strongly stained but unidentified bands are marked with D, E, and F. Molecular weight markers are indicated in the left margins. (B) Determination of the IgE‐binding capacity of the recombinant allergens rUlo c 1, rUlo c 2, and rUlo c 6 by ELISA using sera from mold‐allergic patients. Results are displayed as mean OD values, and standard deviations from two technical replicates are indicated as error bars in the bar charts. The red line represents the cutoff value, which was calculated from the mean OD values plus two times the standard deviation of sera from three non‐allergic patients. Sera that gave OD values above 3.0 are marked with an asterisk (*). (C) Inhibition immunoblot: Blotted proteins of *U. chartarum* protein extracts were exposed to pools of sera (rUlo c 1: sera 25–27, 42, and 78; rUlo c 2: sera 15, 28–32, and 57; rUlo c 6: sera 3–4, 13–14, 28–31, 34–36, and 58–61) that had been pre‐incubated with rUlo c 1, rUlo c 2, or rUlo c 6 or, for control purposes, with buffer only. Molecular weight markers are indicated in the left margins. Arrows point to reduction in IgE binding to natural Ulo c 1, Ulo c 2, and Ulo c 6 after pre‐incubation with the recombinant proteins

Peptide mass fingerprinting allowed to identify a 15 kDa *U. chartarum* protein (A in Figure 1A) as an Alt a 1‐homologous protein, a 41 kDa protein (B) as formate dehydrogenase, a protein not yet known as an allergen, and a 48 kDa protein (C) as an Alt a 6‐homologous enolase. The proteins were designated Ulo c 1^a^ (A), Ulo c 2^b^ (B), and Ulo c 6^c^ (C). The IgE‐reactive protein bands D, E, and F could not yet be identified by mass spectrometry. cDNAs coding for the identified allergens was generated and cloned into a bacterial expression vector, and recombinant proteins were produced in *Escherichia coli*. Circular dichroism spectroscopy revealed that the recombinant allergens contain considerable secondary structures. In case of rUlo c 1, mainly beta‐sheet structures (minimum of far‐UV spectrum at 213 nm) were found, whereas predominantly alpha‐helical structures (minima at 207 nm and 220 nm) were detected for rUlo c 2 and rUlo c 6 (Figure S2).

ELISAs performed with sera from mold‐allergic patients showed the IgE‐binding capacity of the three recombinant allergens, with rUlo c 1 displaying the highest levels of IgE reactivity (Figure 1B). Furthermore, IgE inhibition immunoblots, where nitrocellulose‐blotted protein extracts from *U. chartarum* were exposed to serum pools that had been pre‐incubated with the recombinant proteins, showed that the recombinant allergens were able to completely inhibit patients' IgE binding to their natural counterparts (Figure 1C). This indicates that the recombinant allergens represent well‐folded proteins which contain all the IgE‐binding epitopes present in their natural counterparts and suggests that rUlo c 1, rUlo c 2, and rUlo c 6 could be used as tools for in vitro diagnosis of *U. chartarum* sensitization.

The prevalence of the molecules' IgE reactivity was analyzed by ELISA using sera from 85 individuals sensitized to different mold species (Figure S3). rUlo c 1 was recognized by 58% of the patients, indicating that this molecule represents a major mold allergen, whereas rUlo c 2 was recognized by 43% and rUlo c 6 by 40% of the patients, suggesting minor mold allergens.

Sequence comparison of the three *U. chartarum* allergens with homologous *A. alternata* proteins revealed protein sequence identities of 89% between Ulo c 1 and Alt a 1, 99% between Ulo c 2 and its homologous *A. alternata* protein, and 100% between Ulo c 6 and Alt a 6. The high sequence homology between Ulo c 1 and Alt a 1 and the suggested presence of Alt a 1‐homologous proteins in other species of the Pleosporaceae family^10^ prompted us to investigate the potential cross‐reactivity between rUlo c 1 and rAlt a 1 in IgE inhibition immunoblots. As depicted in Figure 2A, pre‐incubation of patients' sera with one allergen always significantly reduced or even abolished IgE binding to the other molecule, whereas IgE binding was not reduced by pre‐incubation of the sera with the irrelevant respiratory allergen rBet v 1. This reduction in IgE binding obtained with rAlt a 1 and rUlo c 1 was comparable to the reduction obtained by self‐inhibition and indicates that the two allergens share conserved IgE epitopes. Furthermore, both recombinant allergens, rUlo c 1 and rAlt a 1, induced a dose‐dependent expression of CD63 in basophils from six of the eight analyzed mold sensitized individuals (Figure 2B), which provided evidence for the molecules' biological activity.

**FIGURE 2:**
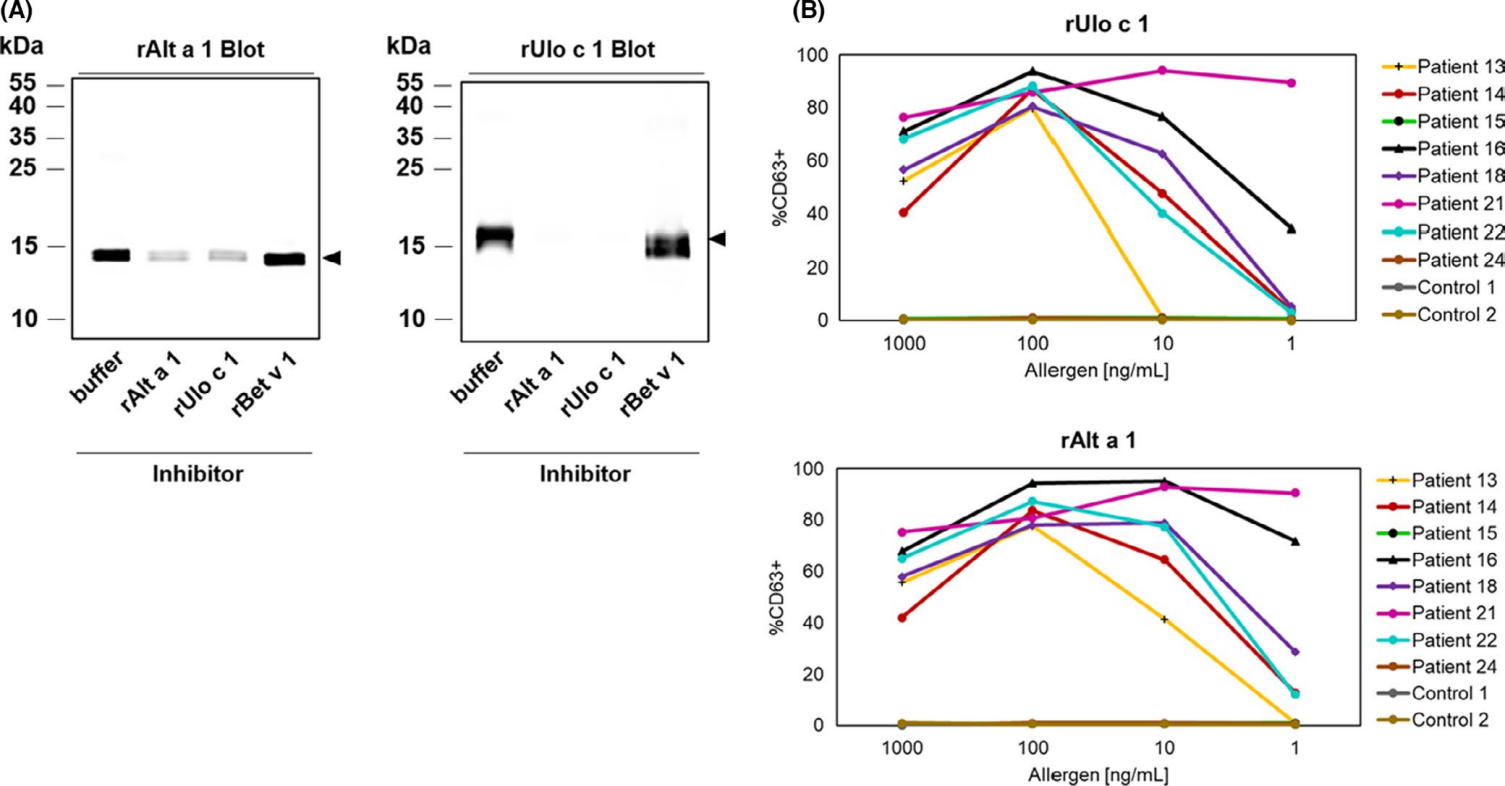
IgE cross‐reactivity between the major fungal allergens rUlo c 1 and rAlt a 1. (A) Two micrograms of the recombinant allergens were separated by SDS‐PAGE, blotted onto nitrocellulose, and exposed to a pool of sera (25–27, 42, and 78) from mold‐allergic patients, which had been pre‐incubated with rUlo c 1 or Alt a 1 or, for control purposes, with the non‐relevant allergen rBet v 1 or with buffer only. Molecular weight markers are indicated in the left margins. (B) Basophil activation of rUlo c 1 and rAlt a 1 was determined by measuring the expression of CD63 by flow cytometry after incubation of the whole blood from eight *Alternaria*‐sensitized patients (patients 13–16, 18, 21, 22, and 24) and from two non‐atopic control individuals (control 1 and 2) with increasing allergen concentrations. The percentage of CD63‐positive basophils (*y*‐axis), based on the total amount of basophils, is displayed against the concentration of the applied allergen (*x*‐axis)

Analysis of the release of allergens from allergen sources can provide useful information about the potential exposure to these allergens. Interestingly, our investigations on the release kinetics of Ulo c 1 and Ulo c 6 from *U. chartarum* and Alt a 1 and Alt a 6 from *A. alternata* showed that the allergens were all immediately released from the spores (Figure S4). These findings suggest that rapid allergen elution might also occur when spores reach mucosal surfaces, where they can then elicit allergic reactions.

In conclusion, in this study we provide evidence that *U. chartarum* represents an important, so far underestimated, allergen source that shows at least partial cross‐reactivity to the evolutionarily related species *A. alternata*. Owing to the fact that in contrast to the outdoor mold *A. alternata*, *U. chartarum* is an important component of the indoor environment, it is especially important to raise the awareness that *U. chartarum* represents a potential cause of respiratory allergic diseases and to include the species into routine allergy diagnosis. We identified the species' first allergens, Ulo c 1, Ulo c 2, and Ulo c 6, and produced them as IgE‐reactive recombinant molecules. These recombinant allergens will expand the repertoire of fungal allergens available for fungal allergy diagnosis, thus improving diagnosis' specificity and sensitivity.

The description of the used methods can be found in the supporting information.

## CONFLICT OF INTEREST

SP, PS, MR, MP, RMMC, CSB, KN, ERF, MFT, KS, and IS have nothing to disclose. SQ reports personal fees and non‐financial support from GSK, personal fees and non‐financial support from AstraZeneca, personal fees and non‐financial support from Sanofi, personal fees and non‐financial support from Novartis, personal fees and non‐financial support from Mundipharma, personal fees and non‐financial support from Teva, and personal fees and non‐financial support from Allergy Therapeutics, outside the submitted work.

## Supporting information

Figure S1

Figure S2

Figure S3

Figure S4

Table S1

Supplementary Material

## Footnotes

^a^
NCBI Nucleotide Sequence Accession Number: MH716394.

^b^
NCBI Nucleotide Sequence Accession Number: MN953052.

^c^
NCBI Nucleotide Sequence Accession Number: MH910061.
