# Supplementary figures and images for: Identification of Ulocladium chartarum as an important indoor allergen source
Source: Allergy. 2021 Jul 28;76(10):3202–6. doi: 10.1111/all.14999 (PMC9290848; doi:10.1111/all.14999)

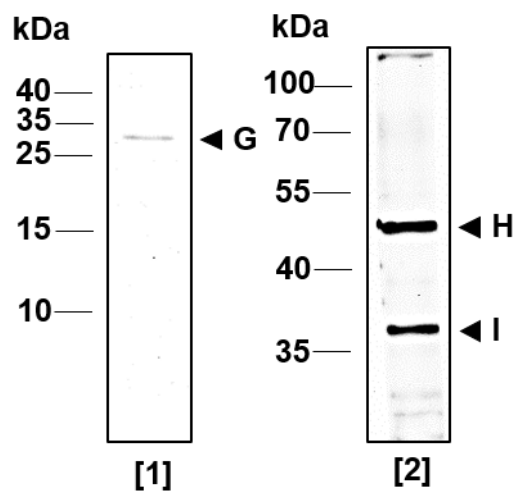

Supplement: Supplementary file 1 — Figure S1 [file ALL-76-3202-s003.pdf]

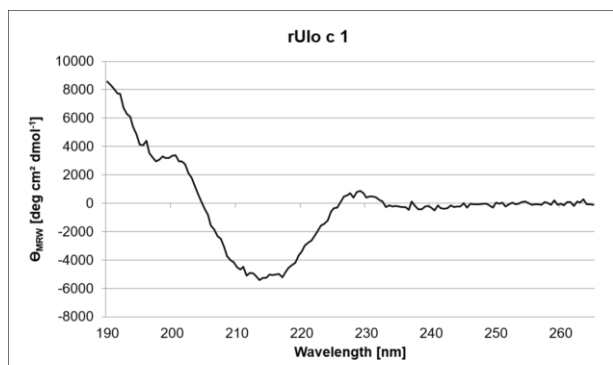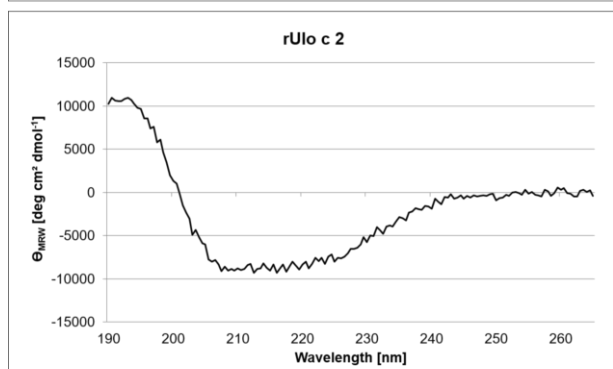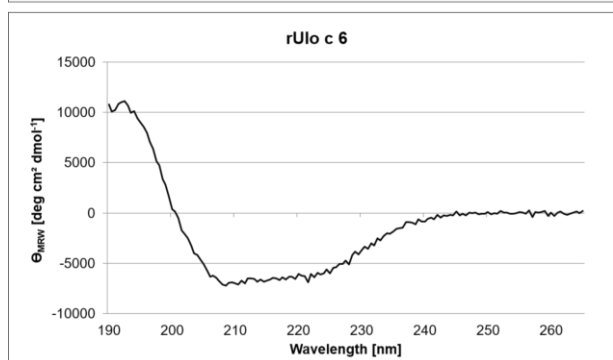

Supplement: Supplementary file 2 — Figure S2 [file ALL-76-3202-s002.pdf]

*Ulocladium chartarum*

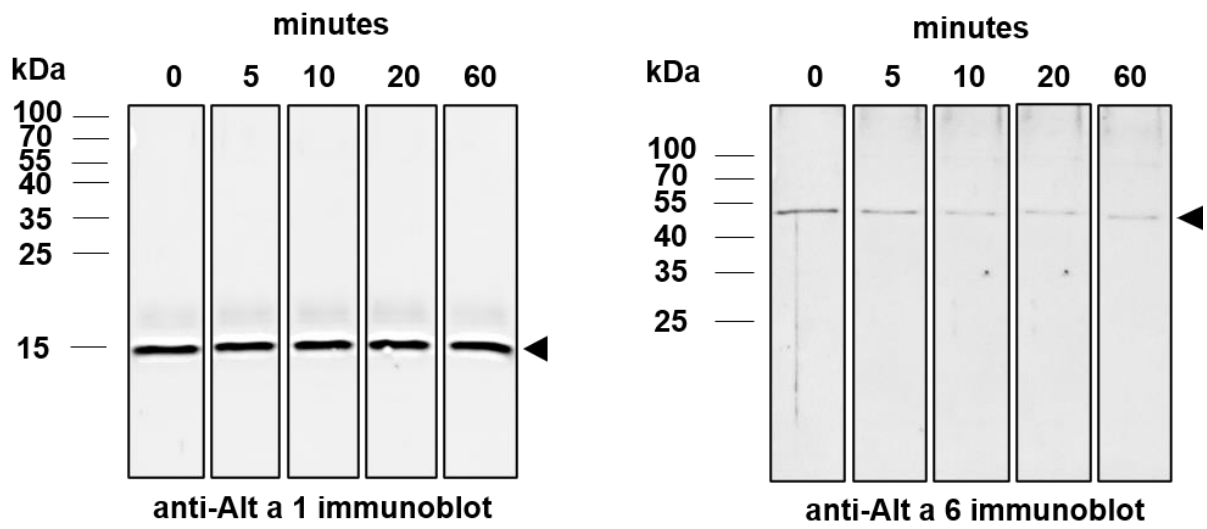

*Alternaria alternata*

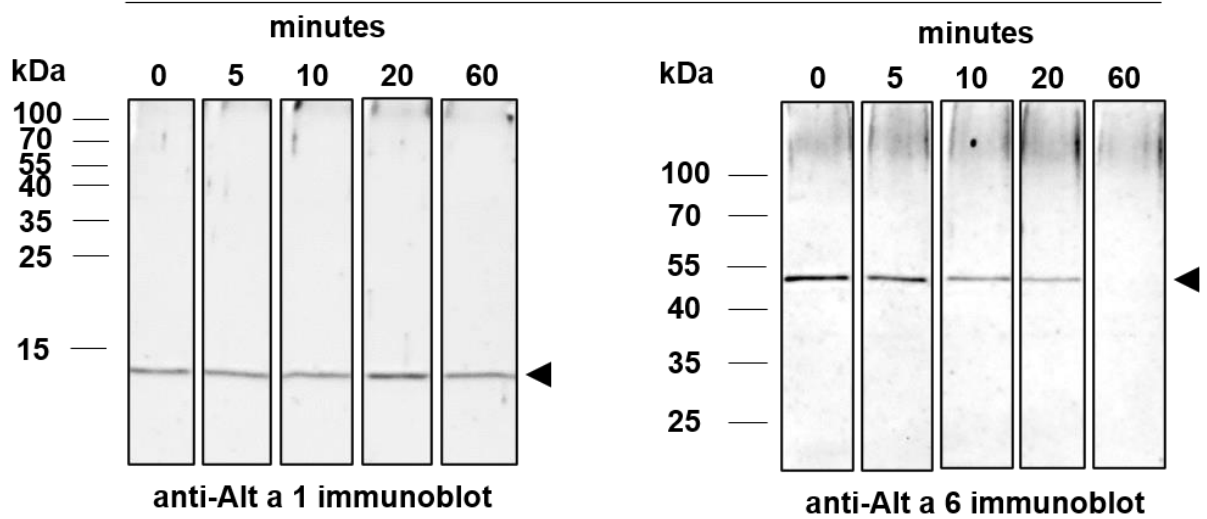

Supplement: Supplementary file 4 — Figure S4 [file ALL-76-3202-s005.pdf]
