# Supplementary material for: Identification of Ulocladium chartarum as an important indoor allergen source
Source: Allergy. 2021 Jul 28;76(10):3202–6. doi: 10.1111/all.14999 (PMC9290848; doi:10.1111/all.14999)
Supplement: Supplementary file 3 — Figure S3 [file ALL-76-3202-s006.pdf]

Patient number	rUloc1	rUloc2	rUloc6	Patient number	rUloc1	rUloc2	rUloc6
1				45			
2				47			
3				48			
4				49			
5				50			
6				51			
7				52			
8				53			
9				54			
10				55			
11				56			
12				57			
13				58			
14				59			
15				60			
16				61			
17				62			
18				63			
19				64			
20				65			
21				66			
22				67			
23				68			
24				69			
25				70			
26				71			
27				72			
28				73			
29				74			
30				75			
31				76			
32				77			
33				78			
34				79			
35				83			
36				84			
37				85			
38							
39							
40							
41							
42							
43							
44							
