# Supplementary material for: Identification of Ulocladium chartarum as an important indoor allergen source
Source: Allergy. 2021 Jul 28;76(10):3202–6. doi: 10.1111/all.14999 (PMC9290848; doi:10.1111/all.14999)
Supplement: Supplementary file 5 — Table S1 [file ALL-76-3202-s001.pdf]

Table S1. Summary of demographic and serological data of mold-allergic patients grouped based on their IgE-reactivity profiles (n = 85). Total IgE (in kU/L) and specific IgE (sIgE; in kUA/L) against different mold species were determined by ImmunoCAP analysis.

Patient numbers	Allergen source	Age (yr)	Total IgE [kU/L]	sIgE mold [kUA/L]
1-27	*Alternaria*	4-83	25-646	1.54-66.60
28-38	*Alternaria*<br>*Cladosporium*	7-36	108-2229	*Alternaria*: 3.50 - >100<br>*Cladosporium*: 0.89-80.10
39; 44	*Alternaria*<br>*Aspergillus*	75; N/A	862; N/A	*Alternaria*: 1.92-3.62<br>*Aspergillus:* 0.01-3.99
40-41	*Alternaria*<br>*Aspergillus*<br>*Penicillium*	35-48	121-1679	*Alternaria*: 01.13-6.00<br>*Aspergillus*: 1.06-1.94<br>*Penicillium*: 0.49-0.81
42	*Alternaria*<br>*Epicoccum*	N/A	N/A	*Alternaria*: 3.37<br>*Epicoccum*: 2.01
43	*Alternaria*<br>*Aspergillus*<br>*Cladosporium*	N/A	762	*Alternaria*: 23.3<br>*Aspergillus*: 22.5<br>*Cladosporium*: 5.73
45-56	*Aspergillus*	12-74	185-1874	2.11-19.60
57-70	*Cladosporium*	4-42	220-3625	3.61-23.60
71-72	*Penicillium*	4-13	66-291	4.04-6.30
73	*Fusarium*	N/A	N/A	4.10
74-77	*Aspergillus*<br>*Cladosporium*	10-11	924-984	*Aspergillus:* 4.54-29.00<br>*Cladosporium*: 7.53-24.70
78-80	*Penicillium*<br>*Cladosporium*	N/A	N/A	*Penicillium*: 1.28-9.10<br>*Cladosporium*: 1.42-8.10
81-82	*Penicillium*<br>*Cladosporium*<br>*Aspergillus*	N/A	N/A	*Penicillium*: 6.93-7.72<br>*Cladosporium*: 8.67-8.99<br>*Aspergillus*: 10.80-11.30
83-85			N/A	

Abbreviation: N/A, information not available
