# Supplementary material for: Identification of Ulocladium chartarum as an important indoor allergen source
Source: Allergy. 2021 Jul 28;76(10):3202–6. doi: 10.1111/all.14999 (PMC9290848; doi:10.1111/all.14999)
Supplement: Supplementary file 6 — Supplementary Material [file ALL-76-3202-s004.docx]

Methods

**Patients’ sera**

Sera from mold-allergic patients were obtained from the Department of Allergy, La Paz, University Hospital, Madrid, Spain, from the Hospital Clinic de Barcelona, University of Barcelona, Spain and from the company AbBaltis (FDA-approved; Sittingbourne, United Kingdom). The demographic and serological characteristics of the patients are given in Table S1. Currently, *Ulocladium chartarum* is not included in the routine diagnosis of fungal allergy. Therefore, it was not possible to focus on sera of *U. chartarum* allergic individuals. Instead, patients with a sensitization to at least one of the fungal species used in routine diagnosis were included. All patients agreed to participate in this study and gave informed consent. This study was approved by the ethics committee of the Hospital Clínic de Barcelone (approval number 2011/6605) and of the La Paz University Hospital in Madrid (EK565/2007). We confirm that all methods were performed in accordance with the relevance guidelines and regulations.

**Sodium dodecyl sulfate-polyacrylamide gel electrophoresis, immunoblot and inhibition immunoblot experiments**

Two µg of recombinant proteins or 5 µg of fungal protein extracts were separated on 10%, 12% or 15% sodium dodecyl sulfate-polyacrylamide gels (SDS-PAGEs), as described by Laemmli (S1) and were transferred electrophoretically onto nitrocellulose membranes (0.2 µm; GE Healthcare, Chicago, IL) (S2). For IgE-immunoblots, membranes were blocked with PBS containing 0.5% Tween20 (PBS-T) and were then exposed to pools of sera from mold-allergic patients, or for control purposes, to a pool of sera from three non-allergic individuals or to PBS-T only. These pools contained either 3, 5, 6, 9 or 16 sera (1:10 diluted in PBS-T). For IgE inhibition immunoblots, pools of sera were pre-incubated overnight with 20 µg of rUlo c 1 or rAlt a 1 or, for control purposes, with the unrelated respiratory allergen rBet v 1 (available in the laboratory) or with PBS-T and then proteins, blotted onto nitrocellulose membranes, were incubated with these pre-incubated sera. For the detection of protein-bound human IgEs, a mouse anti-human IgE antibody, conjugated to horseradish peroxidase (HRP), was used as detection antibody (final dilution 1:5,000; Southern Biotech, Birmingham, AL). The blots were developed using the SuperSignal West Pico Plus Chemiluminescent Substrate (Thermo Fisher Scientific, Waltham, MA) according to the manufacturer’s instructions and imaged using the UVP ChemStudio device (Analytik Jena, Germany).

To detect Alt a 1, Alt a 6 and related proteins, nitrocellulose-blotted proteins were incubated with rabbit antisera either directed against a peptide of Alt a 1 (5’-KISEFYGRKPEGTYYNSLG-3’) or against the recombinant Alt a 6 (Charles River Laboratories, Écully, France). A goat anti-rabbit IgG HRP-labelled antibody (Vector Laboratories Inc, Burlingame, CA; diluted 1:10,000 in PBS-T) was used for detection and the blots were developed and imaged as described above for the IgE immunoblots.

**cDNA cloning and recombinant expression of *U. chartarum* allergens**

The full-length cDNAs coding for the *U. chartarum* proteins were generated from *U. chartarum* RNA by RT-PCR. For this, information on the full-length sequences of the Ulo c 1 and Ulo c 2 cDNAs first had to be obtained by 5’/3’-rapid amplification of cDNA ends (RACE) using the SMARTer RACE 5’/3’ Kit (Takara Bio, Kyoto, Japan). Therefore, primers were designed based on the homologous *Alternaria alternata* cDNA sequences of Alt a 1 (NCBI accession number: AY568627) and of the formate dehydrogenase (NCBI accession number: XM_018525579.1). To obtain the 5’- and 3’-end of the Ulo c 1 cDNA the following primers were used: 5’-AACGCATCCTGCCCTGTCACTACCGA-3’ (for 3’-end amplification) and 5’-GCTGTTCTCGCCGCAAGAGTACCAC-3’ (for 5’-end amplification). For the Ulo c 2 cDNA the following primers were used: 5’-GGAAAAGTTCTTCTCGTCCTCTACG-3’ (for 3’-end amplification) and 5’-CTTGCGCTGACCGTACGCCTTGG-3’ (for 5’-end amplification). Primers for full-length cDNA cloning of Ulo c 1 and Ulo c 2 by RT-PCR were then designed based on the sequences of the RACE-PCR-products.

Since enolases are very conserved proteins, the cDNA coding for Ulo c 6 could be obtained by RT-PCR using forward (5’-ACCATCACCAAGATCCACGCCCGC-3’) and reverse (5’-CAAGTTAACGGCAGTCCTGAA-3’) primers designed based on the nucleotide sequence from the homologous *A. alternata* enolase Alt a 6 (NCBI accession number: U82437.2).

The obtained sequences were submitted to GenBank and received the following accession numbers: MH716394 (Ulo c 1), MN953052 (Ulo c 2) and MH910061 (Ulo c 6).

cDNAs coding for Ulo c 1, Ulo c 2 and Ulo c 6 were cloned with C-terminal hexa-Histidine-tags into the bacterial expression vector pET-17b. Following the successful expression of the allergens as soluble proteins in *E. coli*, the molecules were purified under native conditions by affinity chromatography. The protein concentrations were determined with the MicroBCA Protein Assay Kit (Thermo Fisher Scientific) using BSA as a standard.

For control purposes, rAlt a 1 from *Alternaria alternata* was also produced in *E. coli.* For this, the Alt a 1 cDNA (NCBI accession number: AY568627) was generated from *A. alternata* RNA by RT-PCR and was cloned with a C-terminal hexa-Histidine-tag into the bacterial expression vector pET-17b. rAlt a 1 expression and purification were carried out as described for the *U. chartarum* allergens.

**Secondary structure analysis by circular dichroism spectroscopy (CD-spectroscopy)**

The secondary structure of rUlo c 1, rUlo c 2 and rUlo c 6 was evaluated by circular dichroism spectroscopy on a Chirascan Plus Spectrometer (Applied Photophysics, Leatherhead, United Kingdom) in 10 mM sodium phosphate buffer (PH 7.0) at 20°C and a protein concentration of 0.2 µg/µL using a quartz cuvette (Hellma Analytics, Müllheim, Germany) with a path length of 1 mm. CD-spectra were recorded from 190 to 280 nm with a resolution of 0.5 nm and results were the average of three scans. The final spectra were corrected by subtracting the buffer baseline spectrum, obtained under identical conditions, and normalized to the number of peptide bonds by using the extinction coefficient of the measured protein at 205 nm (ε205) (S3). Results are expressed as mean residual ellipticity θ_MRW_ (deg cm² dmol^-1^) at a given wavelength.

**Enzyme-linked immunosorbent assay (ELISA) of recombinant fungal allergens**

The IgE-reactivity of the recombinant *U. chartarum* allergens was analyzed in ELISAs performed with 85 sera from patients sensitized to different mold species. For this, 96-well Nunc MaxiSorp ELISA plates (Thermo Fisher Scientific) were coated with 4 µg/mL of the recombinant proteins, diluted in bicarbonate buffer (pH = 9.6). Wells were blocked with phosphate buffered saline containing 0.5% Tween20 (PBS-T) and were then incubated with sera from mold-allergic patients (1:5 diluted in PBS-T) or, for control purposes, with sera from three non-allergic individuals (1:5 diluted in PBS-T) or with PBS-T only. Bound IgE antibodies were detected with a HRP-labelled goat anti-human IgE antibody (SeraCare Life Sciences Inc., Milford, MA; diluted 1:2,500 in PBS-T) using 1.8 mM of 2,2’-Azino-bis-(3-ethylbenzothiazoline-6-sulfonic acid) diammonium salt (Sigma-Aldrich) in 60 mM citric acid, 77 mM Na_2_HPO_4_(H_2_O)_2_ and 3 mM H_2_O_2_ as a substrate. After 60 minutes of incubation in substrate solution, the optical density of the samples was measured at a wavelength of 405 nm using the Multiskan FC Photometer (Thermo Fisher Scientific). Experiments were always carried out in duplicates and the results are expressed as mean OD-values. The cut-off, used to differentiate positive and negative IgE-reactivity, was calculated from the mean OD-values plus two times the standard deviation of sera from three non-allergic patients.

**Basophil activation test (BAT)**

To test the biological activity of the recombinant molecules, basophil activation tests were performed using the Flow Cast Kit (Bühlmann, Schönenbuch, Switzerland) according to the manufacturers’ instructions. Whole blood from eight mold-sensitized patients and, for control purposes, from two patients with no fungal sensitization, was incubated with increasing concentrations (1 to 1000 ng/mL) of the recombinant allergens. Basophil activation was assessed by detecting the expression of CD63 by flow cytometry (FACS Canto II, Becton Dickinson, Franklin Lakes, NJ). Activated basophils were gated as SSC^low^/CCR3^+^/CD63^+^. At least 500 basophils were measured, and the percentage of CD63-expressing basophils, based on the total amount of basophils, was calculated.

**Allergen Release Kinetics Experiments**

Clones of *U. chartarum* and *A. alternata* were cultured on MEA-plates at 20-25°C for 7 days. Then, 10 mL of a buffer that mimics the natural nasal fluid (160 mM NaCl, 30 mM KCl, 5 mM MgCl_2_(H_2_O)_6_ and 2 mM CaCl_2_(H_2_O)_2_) (S4) was added to the plates and spores were detached using a Drigalski-spatula. The spore solutions were transferred into centrifugation tubes and were incubated under continuous shaking at 20-25°C. Aliquots of 1 mL each were taken after 0, 5, 10, 20 and 60 minutes and were immediately centrifuged to separate the supernatants, containing the released proteins, from the fungal spores. The supernatants were stored at –20°C and the protein concentrations were determined using the method of Bradford with BSA as a standard (S5). For the detection of released allergens, 0.1 µg of the supernatants were separated on 12% or 15% SDS-PAGEs, blotted onto nitrocellulose (as described before) and exposed to the antisera against Alt a 1 or rAlt a 6.

**REFERENCES:**

S1. Laemmli U. Cleavage of structural proteins during the assembly of the head of bacteriophage T4. Nature. 1970;227:680–5.

S2. Towbin H. Staehelin T. Gordon J. Electrophoretic transfer of proteins from polyacrylamide gels to nitrocellulose-sheets: procedure and some applications. Proceedings of the National Academy of Sciences. 1979;76(9):4350-4354.

S3. Kelly SM, Jess TJ, Price NC. How to study proteins by circular dichroism. Biochim Biophys Acta - Proteins Proteomics. 2005;1751(2):119–39.

S4. Burke W. The Ionic Composition of Nasal Fluid and Its Function. Health. 2014;6:720-728.

S5. Bradford MM. A Rapid and Sensitive Method for the Quantitation Microgram Quantities of Protein Utilizing the Principle of Protein-Dye Binding. Anal Biochem. 1976;254:248–54.

**FIGURE LEGENDS:**

**FIGURE S1. IgE-reactivity of *A. alternata* proteins.** Nitrocellulose-blotted proteins from *A. alternata* were exposed to two pools of sera [pool 1: sera 1,2, 45-47 and 74; pool 2: sera 3-4, 13-14, 28-31, 34-36 and 58-61] from mold-allergic patients. IgE-reactive proteins are marked with G, H and I. Molecular weight markers are indicated in the left margins.

**FIGURE S2.** **Structural analyses of recombinant *U. chartarum* allergens.** Circular dichroism spectra were recorded for the recombinant allergens from 190 to 280 nm. Results shown are the average of three scans and are expressed as mean residual ellipticity θ_MRW_ (deg cm² dmol^-1^) at a given wavelength.

**FIGURE S3. IgE-reactivity profiles of mold-sensitized patients to different recombinant allergens.** The binding of IgE antibodies from mold-sensitized patients to rUlo c 1, rUlo c 2 and rUlo c 6 was measured by ELISA and data are summarized in an IgE-reaction map. The optical density was measured in duplicates and the mean OD-value obtained with sera from three non-allergic individuals plus two times the standard deviation was set as a threshold for a positive reaction, indicated here in green. OD-values below the cut-off indicate negative results and are shown in red, whereas grey coloring indicates that the IgE-reactivity was not evaluated.

**FIGURE S4. Ulo c 1, Alt a 1, Ulo c 6 and Alt a 6 are rapidly released from fungal spores.** Proteins, released from *U. chartarum* and *A. alternata* spores after different times of hydration of the spores (0, 5, 10, 20, 60 min), were separated by SDS-PAGE and were blotted onto nitrocellulose membranes. Membranes were either exposed to the anti-Alt a 1 or to the anti-rAlt a 6 antiserum. Molecular weight markers are indicated in the left margins.
